# Role of Fractalkine/CX3CL1 and Its Receptor in the Pathogenesis of Inflammatory and Malignant Diseases with Emphasis on B Cell Malignancies

**DOI:** 10.1155/2014/480941

**Published:** 2014-03-30

**Authors:** Elisa Ferretti, Vito Pistoia, Anna Corcione

**Affiliations:** Laboratory of Oncology, Istituto Giannina Gaslini, 16147 Genova, Italy

## Abstract

Fractalkine/CX3CL1, the only member of the CX3C chemokine family, exists as a membrane-anchored molecule as well as in soluble form, each mediating different biological activities. It is constitutively expressed in many hematopoietic and nonhematopoietic tissues such as endothelial and epithelial cells, lymphocytes, neurons, microglial osteoblasts. The biological activities of CX3CL1 are mediated by CX3CR1, that is expressed on different cell types such as NK cells, CD14^+^ monocytes, cytotoxic effector T cells, B cells, neurons, microglia, smooth muscle cells, and tumor cells. The CX3CL1/CX3CR1 axis is involved in the pathogenesis of several inflammatory cancer including various B cell malignancies. In tumors the interaction between cancer cells and cellular microenvironment creates a context that may promote tumor growth, increase tumor survival, and facilitate metastasis. Therefore the role of the CX3CL1/CX3CR1 has attracted interest as to the development of potential therapeutic approaches. Here we review the different effects of the CX3CL1/CX3CR1 axis in several inflammatory and neurodegenerative diseases and in cancer, with emphasis on human B cell lymphomas.

## 1. Introduction

Chemokines are small cytokines known for their ability to induce migration of cells such as lymphocytes, dendritic cells (DC), macrophages, and stem cells. Based on the cellular context and the site of expression, chemokines can be divided into “inflammatory chemokines,” that are synthesized and promote recruitment of cells during inflammation and “homeostatic chemokines,” that are constitutively expressed in specific tissues where they regulate leukocyte homing [1, 2]. Some chemokines participate both in immune defense during inflammation and in physiological trafficking of resting leukocytes [1, 2]. Moreover, some inflammatory chemokines are crucial components of tumor microenvironment and have a pivotal role in tumor progression, enhancing cancer cell migration to distant organs [3].

Chemokines are structurally characterized by a “chemokine scaffold,” that is, a conserved protein structure, dependent on two disulfide bonds linking cysteine residues. Based on the relative position of their cysteine residues located in the N-terminal region, chemokines can be divided into four subfamilies, CXC, CC, C, and CX3C [1, 2]. CXC chemokines can be further subdivided depending on the presence or absence of an ELR (Glu, Leu, and Arg) amino acid motif. ELR^+^ CXC chemokines attract neutrophils and possess angiogenic properties, whereas ELR^−^ CXC chemokines are angiostatic and attract T and B lymphocytes as well as natural killer (NK) cells [4]. CC chemokines promote the migration of monocytes, DC, lymphocytes, eosinophils, and basophils. Lymphotactin/XCL1 and fractalkine/CX3CL1 are the only members of the C and CX3C chemokine families, respectively. Lymphotactin attracts T and B lymphocytes and NK cells, whereas fractalkine attracts predominantly T and B lymphocytes, NK cells, and monocytes [1, 2].

Chemokines mediate their functions through binding to seven transmembrane G-protein-coupled receptors defined as CXCR, CCR, CR, or CX3CR [1, 2]. Furthermore, some chemokines bind to multiple receptors and some receptors recognize more than one chemokine.

CX3CL1 consists of a chemokine domain linked to a transmembrane domain via an extended mucin-rich stalk of an extracellular domain. The chemokine is synthesized as membrane-anchored form and may be cleaved in the soluble form by different metalloprotease. [5, 6]. The membrane-anchored CX3CL1 form functions as an adhesion molecule promoting retention of leucocytes to endothelial cells under physiological flow conditions [7]. The soluble CX3CL1 form is released following constitutive shedding operated by A Disintegrin And Metalloprotease (ADAM)10, whereas shedding under inflammatory conditions is mediated primarily by ADAM17 [8, 9]. CX3CL1 cleavage is also mediated by the lysosomal cysteine protease Cathepsin S [10]. Soluble CX3CL1 resembles a conventional chemokine exhibiting efficient chemotactic activity for human monocytes, NK cells, T cells, dendritic cells and, as demonstrated by our group, for a subset of germinal center B cells [5, 11]. CX3CL1 expression has been reported in many cell types of hematopoietic or nonhematopoietic origin, such as endothelial and epithelial cells, lymphocytes, neurons, microglial cells, and osteoblasts [12].

CX3CL1-driven chemotaxis and adhesion are mediated by CX3CR1 that is expressed on different cell types such as NK cells, CD14^+^ monocytes, cytotoxic effector T cells, B cells, neurons, microglia, smooth muscle cells, and tumor cells [11, 13–15]. CX3CL1 is involved in leukocyte recruitment associated with numerous inflammatory disorders and in tumorigenesis process in which the chemokine show pro- and antitumoral properties. The different roles of CX3CL1 make it an attractive candidate for the development of therapeutic strategies.

This review will summarize the multiple roles of the CX3CL1/CX3CR1 axis in the pathogenesis of inflammation and cancer.

## 2. CX3CL1 in Inflammation

Chemokines and adhesion molecules provide signals for trafficking, adhesion, and migration of leukocytes at sites of injury and inflammation [16]. In this context, CX3CL1 promotes the accumulation of immune cells that express CX3CR1, generating a vascular gateway for cytotoxic effector cells and being detrimental in several inflammatory diseases [6, 17, 18].

Increased levels of soluble CX3CL1 have been detected in serum, bronchoalveolar lavage fluids, and supernatants from airway smooth muscle cells, lung endothelium, and airway epithelium of allergic asthma and rhinitis patients. Both high secretion of CX3CL1 and upregulation of CX3CR1 function by naïve and memory CD4^+^ T cells play a critical role in the recruitment of inflammatory cells after allergen stimulation [19, 20]. It has been demonstrated that transfer of CD4^+^ T cells from wild type mice into CX3CR1 deficient mice restores the clinical features of asthma, highlighting the therapeutic potential of the CX3CL1/CX3CR1 axis [21].

Rheumatoid arthritis (RA) is a chronic joint disease characterized by massive infiltration of inflammatory cells into multiple joints, leading to hyperplasia of synovium and destruction of cartilage and bone [22]. Previous studies have defined the role of CX3CL1 in pathogenesis of RA and other chronic diseases such as polymyositis and dermatomyositis [23–26]. CX3CL1 has been detected on fibroblast-like synoviocytes and endothelial cells in RA synovium where it contributes to the accumulation of CX3CR1^+^ T cells, macrophages, and dendritic cells. Following interaction of CX3CL1 with its receptor, these latter inflammatory cells adhere to endothelial cells, migrate into the synovium, and secrete cytokines [23–26]. In a murine model of collagen-induced arthritis (CIA), inhibition of CX3CL1 attenuated clinical symptoms, ameliorated histopathological features, and reduced joint infiltration of inflammatory cells [27].

Similarly to human RA, polymyositis and dermatomyositis are characterized by chronic muscle inflammation with infiltration of CX3CR1^+^ cytotoxic T cells and macrophages, recruited by CX3CL1 expressing vascular endothelial cells and other inflammatory cells [28]. In experimental autoimmune myositis, the treatment with anti-CX3CL1 antibody reduced the numbers of muscle infiltrating cells and ameliorated histological inflammatory lesions pointing to the potential therapeutic role of CX3CL1 inhibition and/or CX3CL1/CX3CR1 block in the treatment of RA and inflammatory myopathies [28].

Evidence for a pivotal role of CX3CL1 in cardiovascular disease has been provided. Atherosclerosis is a disease affecting arterial blood vessels as the result of fatty materials accumulation such as cholesterol [29]. Monocytes-derived foam cells are the hallmark in both early and advanced atherosclerotic lesions and evidence indicates that chemokines play important roles in directing migration of these cells from the blood to the vessel wall [29, 30]. In this respect, high levels of CX3CL1 have been found in vascular smooth muscle cells (VSMCs) of coronary atherosclerotic plaques, whereas mononuclear cells and vascular endothelium express CX3CR1 [31, 32]. The CX3CL1 membrane-bound form promotes cell-cell interactions, whereas the soluble form, cleaved by the cysteine protease Cathepsin S and expressed by VSMCs, regulates the adhesion and capture of circulating monocytes to the sites of atherogenesis [31]. Genetic deletion of CX3CL1 or its cognate receptor dramatically reduces monocyte recruitment in the artery wall and the subsequent development of lesions in murine models of atherosclerosis, suggesting that the chemokine/receptor axis represents an attractive therapeutic target for clinical trials in cardiovascular disease [33]. Recent studies in mice show that treatment with a CX3CR1 antagonist induces potent inhibition of atherosclerotic lesions [34].

An important role for the CX3CL1/CX3CR1 axis has also been demonstrated in renal diseases such as glomerulonephritis, tubulointerstitial nephritis, pyelonephritis, and renal allograft rejection. Upregulation of CX3CL1, expressed preferentially on the apical membrane of renal tubular epithelial cells, has been reported in glomerulonephritis where CX3CL1 acts as a chemoattractant and adhesion molecule [35, 36]. Indeed the apical CX3CL1, firmly anchored to the membrane, facilitates recruitment and retention of the majority of CX3CR1^+^ leukocytes that infiltrate the kidney during glomerulonephritis or other nephropathies [35, 36].

Both CX3CL1 and CX3CR1 are upregulated in patients with chronic liver disease and associated with the severity of liver fibrosis. An increased expression of ADAM10 and ADAM17 by hepatic stellate cells (HSC) has been demonstrated in these patients. Shedding of CX3CL1 by the two metalloproteases facilitates the recruitment and adhesion of CX3CR1^+^ inflammatory cells and the paracrine stimulation of HSC in liver disease [37, 38].

The CX3CL1/CX3CR1 axis plays also important roles in inflammatory bowel diseases. In patients with Crohn's disease, there is a significant increase of CX3CL1 mRNA expression in inflamed lesions compared to noninflamed colonic mucosa suggesting a relevant impact of this chemokine in intestinal inflammation [39]. Experiments in mice demonstrate that intestinal lamina propria (LP) macrophages express high levels of CX3CR1 [40]. Thus, deletion of CX3CR1 resulted in a significant reduction of macrophage recruitment to LP with a decreased translocation of bacteria to mesenteric lymph nodes and their ability to take up pathogens. These findings point to CX3CR1 as a specific marker for LP macrophages and a critical component in maintaining LP macrophage homeostasis [40].

In recent years, increasing evidence for an inflammatory component in age-related macular degeneration has been demonstrated. Under physiological conditions, CX3CL1 is constitutively expressed in retina and retinal pigment epithelium, whereas microglia cells express CX3CR1 [41]. Dysfunction in CX3CL1/CX3CR1 signaling leads to accumulation in the subretinal space of both inflammatory macrophages and microglia cells that participate to the development of retinal degeneration [42].

Recently, two common single-nucleotide polymorphisms (SNPs) located in the open reading frame of the human CX3CR1 gene have been described, namely T280M and V249I [43]. These two variants are in strong linkage disequilibrium. In the T280M variant, a methionine replaces a threonine residue whereas a valine is replaced by an isoleucine in the V249I variant. The frequencies of these two variants are significantly associated to a lower risk of inflammatory diseases such as atherosclerosis, coronary artery disease, susceptibility to human immunodeficiency virus infection, age-related macular degeneration, and Crohn's disease [44–49].

Table 1 summarizes the role of CX3CL1/CX3CR1 axis in the different inflammatory disorders.

## 3. Neuroinflammatory versus Neuroprotective Roles of CX3CL1

CX3CL1 is abundantly expressed by neurons in the central nervous system (CNS), where it regulates the communication between neurons, glia, and microglia cells, sustaining the normal microglial activity through interaction with its receptor. CX3CR1 is also highly expressed by microglia, astrocytes, and hippocampal neurons in the CNS [50, 51]. Thus, CX3CL1 serves as a neuronal regulatory protein controlling microglia activation under physiological conditions. Depending on type of CNS injury, the CX3CL1/CX3CR1 axis plays a different role in neurodegeneration versus neuroprotection.

Alzheimer's disease (AD) is a progressive neurodegenerative disorder characterized by alteration of neurons and loss of memory and cognitive function. The pathological hallmarks of AD are massive deposits of amyloid beta (A*β*) in the brain leading to microglia activation and neuroinflammation. Proinflammatory chemokines, cytokines, and neurotoxins contribute to neuronal degeneration observed in AD. In mice overexpressing amyloid precursor protein, CX3CR1 depletion results in a reduction of A*β* deposition and amelioration of the disease [52, 53]. In contrast, other experimental AD models show that CX3CR1 deficiency enhances microglia activation and worsens memory and cognitive functions [54, 55] suggesting that this discrepancy may be related to the different mouse models used in these studies.

Depletion of dopaminergic neurons in the substantia nigra characterizes Parkinson's disease (PD). Recent evidence suggests that microglia activation is a key component contributing to this dopaminergic degeneration [56, 57]. High CX3CL1 levels correlate with the progression and severity of the disease as demonstrated in CX3CL1 or CX3CR1 deficient mice which show an increased brain neurodegeneration [58, 59]. In a mouse model of PD, the two forms of endogenous CX3CL1 have been detected with different effects on disease progression. The soluble form exhibits neuroprotective capability since it decreases dopaminergic neuron loss, reduces impairment of motor coordination, and ameliorates microglial activation and proinflammatory cytokine release. In contrast, the membrane-bound form is not neuroprotective but mediates proinflammatory functions [58]. Further studies are necessary to expand the knowledge on the role of both forms in* in vivo* models of neuroinflammation and neurodegeneration.

Human immunodeficiency type I (HIV) infection causes HIV-associated neurocognitive disorders (HAND) including HIV-dementia [60]. The above-discussed balance between neuroprotective and neurotoxic roles of CX3CL1 is also observed in HIV dementia. On the one hand, CX3CL1 has a clear protective effect since its upregulation and secretion inhibit apoptosis of hippocampal neurons induced by neurotoxic viral proteins [61, 62]. On the other hand, CX3CL1 is involved in neuronal damage through its activity on microglia cells that secrete proinflammatory cytokines. Moreover, microglia and macrophages expressing CX3CR1 are in turn activated and recruited to the neuronal injury sites, thus amplifying the inflammatory response [51, 63].

The role of CX3CL1/CX3CR1 axis in the different neurological disease is summarized in Table 1.

## 4. CX3CL1/CX3CR1 in Cancer

The role of the CX3CL1/CX3CR1 axis in cancer pathogenesis has long been discussed. The expression of CX3CR1 in NK cells and some T cells subsets has provided the rationale to exploit the role of the chemokine/receptor in cancer immunotherapy setting the stage for potential therapeutic approaches. The dual function of CX3CL1 as chemoattractant for leukocytes and adhesion molecule for tumor cells that express the receptor may explain the discrepancies reported in the literature regarding the tumorigenesis process [17].

Recently, expression and function of CX3CL1 and CX3CR1 have been demonstrated in glioma tumors irrespectively of their histotype and clinical severity. Gliomas are the most common brain tumor in humans, characterized by high invasion rate and diffuse infiltration of the CNS. Gliomas include heterogeneous tumors classified according to the pathological characteristics as astrocytomas, oligodendrogliomas, and glioblastoma [64, 65]. CX3CL1 negatively regulates glioma cell invasiveness by promoting tumor cell aggregation when expressed as transmembrane protein. The induction of cell to cell contact by CX3CL1 prevented detachment of tumor cells from the tumor aggregate that is required for the invasion process [64, 65]. Treatment of glioma cells with recombinant transforming growth factor (TGF)-beta 1 reduced CX3CL1 expression at mRNA level, facilitating glioma cell detachment and dispersion [66]. Moreover, Erreni and colleagues demonstrated that glioblastoma cancer stem cells and progenitor cells express both CX3CL1 and CX3CR1, indicating that this axis operates early in tumorigenesis process [65].

Neuroblastoma (NB) is the most common extracranial tumor of childhood that originates from the neural crest and presents with metastases at diagnosis in about half of patients. This tumor regresses spontaneously in infant, whereas children older than one year have poor prognosis. The bone marrow is the preferred site of NB metastases, but also bone, liver, and skin are frequently involved [67, 68]. Previous studies have demonstrated that several human NB cell lines express functional CX3CR1 and CX3CL1. Soluble CX3CL1 stimulates NB cells that express CX3CR1 to transmigrate through CX3CR1^+^/CX3CL1^+^ human bone-marrow endothelium, suggesting a prometastatic effect of the chemokine/receptor axis [69]. In other studies, delivery of the CX3CL1 gene into NB cell lines induces an effective antineuroblastoma immune response mediated by NK cells and T lymphocytes. This chemokine gene therapy is amplified by anti-GD2 antibody/IL-2 fusion protein and may provide a promising approach for neuroblastoma [70].

Tumors of nonneuronal origin, for example, prostate, pancreas, and breast cancer, show overexpression of CX3CR1 that regulates adhesion and migration of tumor cells to metastatic sites [15]. In prostate cancer, Shulby and colleagues demonstrated* in vitro* that CX3CL1 and its receptor direct prostate cancer cells to the bone marrow and guide their preferential migration towards human osteoblasts. Bone marrow endothelial cells express the membrane form of CX3CL1, that is cleaved by osteoblasts and released as soluble molecule able to attract prostate cancer cells [71]. Not only osteoblasts but also mesenchymal stromal cells secrete soluble CX3CL1 in the acellular fraction of bone marrow. This generates a gradient that attracts CX3CR1-bearing cells from the blood to the bone marrow [72]. These findings support the rationale for the use of CX3CR1, CX3CL1, and metalloproteases responsible for its cleavage as potential therapeutic targets for prostate cancer.

Pancreatic ductal adenocarcinoma (PDAC) represents a highly aggressive tumor characterized by rapid progression and chemoresistance. The peculiarity of this tumor is tropism for local peripheral nerves that is a major cause of local tumor recurrence [73]. Tumor cells from PDAC patients strongly expressed CX3CR1 that mediates migration to CX3CL1 constitutively expressed by neural cells [74]. The high frequency of CX3CR1 and the marked perineural invasion in PADC patients supported the concept that CX3CR1 may have an important role in the spreading of pancreatic cancer cells along peripheral nerves and in predicting early tumor relapse after surgery. To confirm this hypothesis, Marchesi and colleagues demonstrated that a tumor infiltrated in peripheral nerves was present only in mice injected with CX3CR1-transfected PADC cells [74]. In conclusion, the CX3CR1/CX3CL1 axis could represent a potential therapeutic approach using antagonists to CX3CR1 capable to inhibit tumor neurotropism in PADC.

In epithelial ovarian carcinoma (EOC) CX3CL1 is produced by epithelial cells and promotes malignant cell proliferation [75]. CX3CR1 is virtually absent in normal ovarian surface epithelium and accumulates during the course of tumorigenesis process [76, 77]. The interaction between CX3CL1 and CX3CR1 facilitates cell migration and cell adhesion between EOC cells and peritoneal mesothelial cells, contributing to EOC cell proliferation [77].

Breast cancer is the most common malignancy among women. Metastasis is the main cause of morbidity and mortality associated with this tumor [78, 79]. Several experimental models have analyzed the influence of the CX3CR1/CX3CL1 axis in the biology of breast cancer and the possible therapeutic targeting of CX3CL1. Normal and malignant breast tissues express CX3CR1 but its overexpression increases the ability of tumor cells to migrate to skeleton and brain where bone stromal cells and neurons release soluble CX3CL1 [79, 80]. These findings support the protumoral role of CX3CR1 in breast cancer dissemination. In this view, CX3CL1^−/−^ transgenic mice inoculated with cancer cells show a strong reduction of skeletal dissemination compared to wild-type animals [79]. In addition, relationships between CX3CL1 expression and patient prognosis are demonstrated in breast cancer. High levels of CX3CL1 correlate with good prognosis and are identified as prognostic factors for disease survival [81]. These antitumoral effects are due to immunological mechanisms since the chemokine enhances the recruitment of CD8^+^ T cells, CD1a^+^, DCs, and NK cells, inducing both innate and adaptive immunity [81]. Therefore, CX3CL1 expression could be considered an essential biomarker for predicting prognosis and identify patients eligible for immunomodulating therapy in breast cancer.

CX3CL1 is considered a prognostic biomarker also for patients with colorectal cancer and hepatocellular carcinoma. Colorectal cancer (CRC) is the second cause of cancer death since one half of all patients develop metastasis especially in the liver and lung [82]. High CX3CL1 expression has been found to be a marker of better prognosis in CRC [83]. In murine models soluble CX3CL1, produced by colon cancer cells, drastically reduced their metastatic potential and growth in the target organs through immune mechanisms [84]. The antitumor effects of CX3CL1 are mostly related to attraction of cytotoxic effector T lymphocytes and NK cells.

Hepatocellular carcinoma (HCC) usually follows chronic infectious hepatitis resulting in liver fibrosis [85]. Expression of both CX3CL1 and CX3CR1 influences the clinical features and prognosis in patients with HCC since high expression of the chemokine/receptor axis correlates with better prognosis and tumor differentiation [86]. In a murine model, Tang and colleagues showed that transfer of the CX3CL1 gene into tumor cells elicited tumor-specific cytotoxic T cells and increased production of IL-2 and IFN-*γ* in tumor tissue leading to inhibition of HCC growth [87].

In this respect, CX3CL1 may be considered a chemokine suitable for immunoprevention or gene therapy in colorectal cancer, hepatocellular carcinoma, and, more recently, in gastric adenocarcinoma where its expression correlates with induction of both innate and adaptive immunity [88].

In conclusion, two different mechanisms operate in cancer, leading to protumoral or antitumoral effects of CX3CL1. On the one hand, CX3CL1 stimulates a strong immune response with the recruitment of NK cells and tumor-specific T cells. On the other hand, CX3CL1 plays a pivotal role in tumor angiogenesis, as well as in adhesion and migration of cancer cells, reinforcing their ability to spread and metastasize.  Table 3 summarizes the role of CX3CL1/CX3CR1 axis in cancer.

## 5. CX3CL1/CX3CR1 Axis in B Cell Malignancies

We have previously demonstrated that CX3CL1 is expressed on human naïve, germinal center (GC), and memory B cells, the major B cell subsets from secondary lymphoid tissues [11]. In these sites, soluble CX3CL1 produced by T follicular helper and follicular dendritic cells was found to control both the trafficking of human CX3CR1^+^ centrocytes present in the GC light zone and their* in vivo* survival and differentiation [11].

Recently, a novel role for CX3CR1/CX3CL1 axis has been also delineated for B cell malignancies. Our group demonstrated the involvement of CX3CR1 in the crosstalk between neoplastic B cells and tumor microenvironment of patients with chronic lymphocytic leukemia (B-CLL) [89]. B-CLL cells were found to coexpress CX3CR1 and membrane-anchored CX3CL1 on the cell surface and constitutively release the soluble form of the chemokine. Only a fraction of B-CLL samples was found to be attracted* in vitro* by CX3CL1. Leukemic B cells upregulated CXCR4 upon incubation with CX3CL1 and this was paralleled by increased chemotaxis to CXCL12. Nurse-like cells generated from CLL patient blood coexpressed CX3CR1 and CX3CL1, but only a small proportion of them migrated* in vitro* to CX3CL1 that is not secreted by nurse-like cells. Based upon these findings, we have proposed a model whereby the CX3CR1/CX3CL1 axis may contribute to interactions between CLL cells and tumor microenvironment by increasing CXCL12-mediated attraction of leukemic cells to NLC and promoting directly adhesion of CLL cells to NLC [89].

In studies by other groups, CX3CR1 expression was investigated in different types of B cell lymphomas by reverse transcriptase-polymerase chain reaction (RT-PCR), immunohistochemistry, and flow cytometry. B cell lymphomas include about 80% of the malignant lymphomas and comprise different subtypes. In particular, diffuse large B cell lymphoma (DLBCL) is the most frequent subtype, representing 30%–35% of all non-Hodgkin lymphomas (NHL). DLBCL has predominant centroblastic morphology, is highly proliferating and invades the GC subverting the physiological microenvironment. Follicular lymphoma (FL) has centrocytic and centroblastic components in different ratios depending on tumor grade and, compared to DLBCL, shows lower proliferative activity and slower invasion of GC by tumor cells. Both DLBCL and FL arise commonly in adults and rarely in children or adolescents [90]. Mantle cell lymphoma (MCL) is a relatively rare type of NHL that displays an aggressive course with a continuous relapse pattern [91]. Marginal zone lymphomas (MZLs) are indolent B cell lymphomas with variable symptoms related to lymphoma location [92]. In particular, extranodal MZLs of mucosa-associated lymphoid tissue (MALT) arise in gastrointestinal tract but may affect every organ in the body. MALT lymphoma infiltrates B cell follicles in the Peyer's patch marginal zone, spreading in the surrounding tissue, and show the same cytological features and immunophenotype as MZLs [93]. DLBCL, FL, MCL, and MALT lymphoma were found to express CX3CR1 at mRNA and protein levels suggesting a functional role for this receptor in the interaction between lymphoma cells and tumor microenvironment [94, 95]. CX3CR1 expression in human FL, MCL, and MZL has been confirmed by our group (Table 2) (unpublished data). In addition, we have demonstrated for the first time that freshly isolated malignant B cells express the membrane-bound form of CX3CL1 (Table 2) (unpublished data). To investigate the functional activity of CX3CR1, chemotaxis experiments utilizing a transwell assay were performed* in vitro*. One-third of MCL samples analyzed in this study migrated to soluble CX3CL1. In contrast, the chemokine did not induce locomotion of FL and MZL cells (Table 2) (unpublished data).

These preliminary results provide evidence that lymphoma B cells show a different migratory behavior in response to CX3CL1 compared to their normal counterparts. Thus, FL cells, which are of GC origin, did not migrate* in vitro* to soluble CX3CL1 that, in contrast, was found by our group to be chemotactic for normal GC B cells [11]. In contrast, a minority of MCL cell fractions migrated to soluble CX3CL1 whereas their normal counterparts, that is, naïve B cells, did not [11].

The role of CX3CL1/CX3CR1 axis in B cell malignancies is shown in Table 3.

## 6. Translational Perspectives

The CX3CL1/CX3CR1 axis plays an important function in the pathophysiology of several inflammatory, infectious, and neurological processes and in various forms of cancers. As apparent from this review, the CX3CL1/CX3CR1 axis promotes inflammation and tumor growth in the majority of disease models discussed. Therefore, therapeutic targeting of this axis represents a promising translational development. In principle, this can be achieved using blocking antibodies to or chemical antagonists of CX3CR1. CX3CL1 shedding may be blocked using chemical antagonists of ADAM10/ADAM 17 or Cathepsin S, although this approach is far from being selective.

So far, no clinical grade CX3CR1 antagonists of any kind have been developed. However, some chemical antagonists have been synthesized that hold promise for future studies.

## Figures and Tables

**Table 1:** Role of CX3CL1/CX3CR1 axis in inflammation and neurodegenerative diseases.

Allergic asthma and rhinitis	CX3CL1 increases recruitment of CX3CR1^+^ CD4^+^ T cells in the airways

Rheumatoid arthritis	CX3CL1 contributes to the accumulation in the synovium of T cells, macrophages, and dendritic cells expressing CX3CR1

Atherosclerotic disease	(i) Membrane-bound CX3CL1 promotes cell to cell interactions (ii) Soluble CX3CL1 directs migration of CX3CR1^+^ monocytes from the blood to the vessel wall

Renal diseases	CX3CL1 supports recruitment and retention of CX3CR1^+^ leukocytes infiltrating the kidney

Chronic liver disease	(i) CX3CL1 facilitates recruitment and adhesion of CX3CR1^+^ inflammatory cells to the liver (ii) CX3CL1 supports paracrine stimulation of hepatic stellate cells expressing CX3CR1

Age-related macular degeneration	Dysfunction in CX3CL1/CX3CR1 signaling promotes accumulation of inflammatory macrophages and microglia cells

Crohn's disease	CX3CL1 sustains homeostasis of macrophages of lamina propria expressing CX3CR1

Alzheimer's disease	(i) CX3CR1 deficiency enhances *β*-amyloid deposition and microglia activation (ii) In other models CX3CR1 depletion results in a reduction of A*β*-deposition

Parkinson's disease	(i) Soluble CX3CL1 exhibits neuroprotective properties decreasing microglial activation and proinflammatory cytokine release (ii) Membrane-bound CX3CL1 is not neuroprotective but mediates proinflammatory functions

HIV infection	(i) Soluble CX3CL1 inhibits apoptosis of hippocampal neurons induced by neurotoxic viral proteins (ii) CX3CL1 is involved in neuronal damage through its activity on microglia that secrete proinflammatory cytokines

**Table 2:** Expression and function of CX3CL1/CX3CR1 axis in human B cell lymphomas.

	CX3CR1 (mean% ± SD)	CX3CL1 (mean% ± SD)	CHEMOTAXIS assay (*n*° migrated cases/*n*° cases)
FL	47.2 ± 12	48.3 ± 9	0/10
MCL	53.8 ± 10	58.9 ± 11	4/14
MZL	64 ± 8	40.2 ± 10	0/9

Expressions of CX3CR1 and CX3CL1 was analyzed on B cells from FL, MCL, or MZL patients by flow cytometry. The results (columns 2 and 3) are expressed as mean percentage positive cells ± SD. Chemotaxis of FL, MCL, and MZL cells was investigated in a transwell assay. In the right column the number (*n*°) of migrated cases to 300 ng/mL CX3CL1 is shown, in respect to the total number of cases analyzed.

**Table 3:** Role of CX3CL1/CX3CR1 axis in cancer.

Gliomas	CX3CL1 negatively regulates glioma cell invasiveness by promoting aggregation of CX3CR1^+^ tumor cells

Neuroblastoma (NB)	(i) Soluble CX3CL1 stimulates CX3CR1^+^ NB cells to transmigrate through CX3CR1^+^/CX3CL1^+^ human bone-marrow endothelium (ii) Deletion of CX3CL1 gene into NB cell lines induces an antitumor immune response mediated by NK cells and T lymphocytes

Prostate cancer	Soluble CX3CL1 attracts CX3CR1^+^ prostate cancer cells to the bone marrow and guides their preferential migration towards human osteoblasts

Pancreatic ductal adenocarcinoma (PDAC)	CX3CR1 mediates migration of PDAC cells to CX3CL1 constitutively expressed by neural cells

Epithelial ovarian carcinoma (EOC)	CX3CL1/CX3CR1 axis facilitates cell migration and cell adhesion between EOC cells and peritoneal mesothelial cells

Breast cancer	(i) CX3CR1 contributes to tumor metastasis to skeleton and brain where bone stromal cells and neurons release soluble CX3CL1 (ii) CX3CL1 induces both innate and adaptive immunity and correlates with good prognosis

Colorectal cancer	Soluble CX3CL1, produced by colon cancer cells, attracts cytotoxic effector T lymphocytes and NK cells showing antitumor effects

Hepatocellular carcinoma	CX3CL1/CX3CR1 axis elicits tumor-specific cytotoxic T cell response and correlates with good prognosis

Gastric adenocarcinoma	CX3CL1 promotes both innate and adaptive immunities

B-chronic lymphocytic leukemia (B-CLL)	CX3CL1/CX3CR1 axis, coexpressed on B-CLL cells, is involved in the interaction between leukemic cells and tumor microenvironment

B cell lymphomas	CX3CL1/CX3CR1 axis, coexpressed on lymphoma cells, may be involved in the interaction between lymphoma cells and tumor microenvironment
